# A Reasoning Hardware Platform for Real-Time Common-Sense Inference

**DOI:** 10.3390/s120709210

**Published:** 2012-07-04

**Authors:** Jesús Barba, Maria J. Santofimia, Julio Dondo, Fernando Rincón, Francisco Sánchez, Juan Carlos López

**Affiliations:** Department of Technology and Information Systems, Computer Engineering School, University of Castilla-La Mancha, Ciudad Real 13071, Spain; E-Mails: mariajose.santofimia@uclm.es (M.J.S.); juliodaniel.dondo@uclm.es (J.D.); fernando.rincon@uclm.es (F.R.); psm1984@gmail.com (F.S.); juancarlos.lopez@uclm.es (J.C.L.)

**Keywords:** common-sense, context reasoning and understanding, FPGA, hardware-acceleration

## Abstract

Enabling Ambient Intelligence systems to understand the activities that are taking place in a supervised context is a rather complicated task. Moreover, this task cannot be successfully addressed while overlooking the mechanisms (common-sense knowledge and reasoning) that entitle us, as humans beings, to successfully undertake it. This work is based on the premise that Ambient Intelligence systems will be able to understand and react to context events if common-sense capabilities are embodied in them. However, there are some difficulties that need to be resolved before common-sense capabilities can be fully deployed to Ambient Intelligence. This work presents a hardware accelerated implementation of a common-sense knowledge-base system intended to improve response time and efficiency.

## 1. Introduction

Nowadays, the hot topic of intelligent systems research is at achieving smart spaces. Over the past ten years Mark Weiser's idea of the invisible computer has been gaining interest and important research efforts have been addressed in that direction. As a consequence, the great concern in this field led the IST Advisory Group to coin the term “*Ambient Intelligence*” [1] for referring to those envisioned environments, where the surrounded, intelligent and intuitive devices are capable of recognizing and responding to arising needs and requirements. Roughly, it can be said that the Ambient Intelligence concept extends the Ubiquitous Computing paradigm by enhancing devices with the required intelligence to support humans in their everyday life activities.

The scenarios described in [1] were supposed to provide a likely glimpse of how computer interactions were expected to be in 2010. However, now that 2010 is over, it is an unquestionable fact that yet there is a gap between the scenarios envisioned by the European Commission and what so far has been reached by the scientific community. This gap poses an urgent need to determine the cause that is preventing Ambient Intelligence from being a reality. To the best of our knowledge the weak point of the Ambient Intelligence paradigm is neither rooted at technology nor at how it is being exploited. The core problem that prevents Ambient Intelligence from being a reality is found at implementing the reasoning capabilities that should lead the environment to respond in accordance to environmental on-going situations. In this respect, the leading cause of the aforementioned gap suggests that research efforts should be addressed to overcome the over-simplistic implementations of cognitive and understanding capabilities that have characterized the proposals presented to date.

The problem of building systems for Ambient Intelligence should be therefore addressed from the same perspective as building intelligent systems. Please, notice that in contrast to expert systems, in which specialized knowledge about a concrete topic is demanded, intelligent systems should count on a great deal of knowledge that support them in dealing with a great variety of problems. Not only is the general knowledge required but also the capability to reason about that knowledge is indispensable. Unfortunately, ineffective means to understand and reason about on-going situations are indeed incompatible with the essence of intelligent systems and systems for Ambient Intelligence. Those systems are expected to exhibit an autonomous and intelligent behavior by understanding and reacting to the activities that are taking place in the supervised environment. These systems should therefore be built upon an application that provides them with a proper means to both manage domain information and generate appropriate responses depending on that information.

Understanding the activities that are being carried out in an Ambient Intelligence environment involves the interpretation of the events that are being captured by the electronic devices deployed in it. Apparently, this activity looks like trivial or a simple task. At least, humans do not seem to have problem in inferring on-going situations given the occurrence of a set of facts. For example, if the light is off, the projector is on, and there are people in the room, it can be easily inferred that a presentation is likely to be going on. However, and in contrast to what it might be expected, computers struggle to success in such endeavors. Cognitive and understanding capabilities are the mechanisms that enable humans to succeed in identifying and recognizing situations given a set of happening events.

The notion of context is at the heart of the Ambient Intelligence paradigm because of its role in narrowing down the meaning of those events that take place in a supervised environment. Also, it helps in determining the most suitable means to react to those situations. However, the task of modeling and reasoning about context yet remains one of the most challenging topics of Ambient Intelligence.

An analysis of how humans succeed in understanding our surroundings brings into light that rather than specific or expert knowledge, the task of understanding the dynamics of a context involves holding a great deal of knowledge about how the world works, or what traditionally has been referred to as *common-sense* knowledge.

The agreement on the key role that common sense plays in building intelligent systems is one of the axiomatic facts of the approach presented here. Efforts are therefore addressed to enacting common-sense knowledge and reasoning capabilities as a prerequisite to the automation of the cognitive and understanding processes demanded in Ambient Intelligence. Nevertheless, Ambient Intelligence peculiarities poses some serious restrictions on response times that the usefulness of the provided responses depends on. In this sense, it is preferable to achieve a “*not-so-good*” yet timely response rather than a complete one that arrives too late to be useful.

In essence, the way how search and inference mechanisms are implemented is what makes the difference between the approaches proposed to date. In this regard, the Scone Knowledge-Base system (http://www.cs.cmu.edu/sef/scone/), which has inspired this work, adopts a marker-passing strategy showing excellent results in minimizing the time employed in search and inference tasks. The fact that the marker-passing mechanism implemented by Scone was ideally devised for massively parallel processing helps in providing very good results regarding the scalability and expressiveness demands.

Despite the good results achieved by Scone, the complexity of the understanding mechanisms involved in interpreting context events poses an arising concern for minimizing latency times. Motivated by this need, this work proposes a hardware implementation of the marker-passing strategy firstly implemented in LISP for Scone. Applied to the reasoning mechanism implemented by Scone, the purpose of this work is to propose a hardware accelerated implementation that minimizes response times and improves efficiency issues.

Our proposal is based on the use of reconfigurable logic devices such as FPGAs (Field Programmable Gate Arrays). The main strength of these devices consists in their ability to support flexible and scalable designs, even performed at run time. FPGAs also offers close to ad-hoc hardware acceleration times without incurring the expenses of tailored silicon architectures. Moreover, the use of massively parallel processor architectures seems to be other alternative to support efficient Artificial Intelligence reasoning tasks. Two scenarios must be considered in that case: (1) the use of conventional microprocessor clusters, and (2) the use of specialized processors for the corresponding application domain, along with built-in distributed local memories. For the first case, scalability is not a problem. However, the memory subsystem turns into the bottleneck, leading to a high rate of cache misses. For the second configuration, the memory bandwidth and latency problem is overcome. On top of this, the presence of specialized processors enables the exploitation of parallelism at all levels and the reduction of response times. However, these architectures usually have a limited number of processing nodes and memory capacity, which becomes a boundary for the size of problems they can tackle.

The architecture described here encompasses the strengths of the aforementioned systems, while at the same it solves the challenging scalability issue by using the reconfigurable technology. It is also worth mentioning that the proposed approach could be potentially integrated in the Scone software system, in a user-transparent manner that would not require any extra work from the user's point of view to make the most out of the hardware facilities.

The rest of this paper is structured as follows. First, in Section 2 a summary of the related work addressing ad-hoc hardware architectures for different computing paradigms in Artificial Intelligence is presented. Special emphasis is made in *common-sense* systems. In Section 2.3, the Scone knowledge-base system is introduced in order to provide the reader with the necessary background upon which the proposed hardware implementation is inspired in. Section 3 is fully dedicated to the hardware implementation details, although some inner aspects of the Scone approach are also provided. For understanding purposes an incremental approach has been adopted, starting up with a description of the basic architecture and operations that are supported. In Section 4, performance results are assessed by comparing the proposed hardware implementation approach with the Scone system, which works as the reference software implementation. Finally, the last section is devoted to drawing the conclusions achieved as a result of the proposed approach.

## 2. Related Work

In this section we intent to present the reader the main references to the most relevant works in each one of the following research fields: application of common-sense knowledge to reasoning automation, contributions of common-sense to context modeling and hardware architectures for artificial intelligence. The first two subsections postulate common sense as one of the mainstream techniques in Artificial Intelligence. The last subsection is a compilation of some examples of ad-hoc hardware architectures built to accelerate Artificial Intelligence algorithms, usually based on graph search and marker propagation.

### 2.1. Automating Common-Sense Reasoning

Automating common-sense reasoning has been one of the primary concerns for researchers in the Artificial Intelligence field. According to E. K. Muller, who in [2] provides a brief history of the common-sense reasoning, first works in this field date from 1956. The main contributions to this field come from authors such as A. Newell, mainly concerned with the cognitive aspect of the Artificial Intelligence, M. Minsky, who has made enormous contributions in the domain of common-sense knowledge representation and reasoning [3,4], and finally D. Lenat, who undertook in 1984 the first real attempt to catalog common-sense knowledge, under the Cyc project [5].

Automating common sense reasoning is a task that requires an expressive-enough language, a knowledge base to store such a large amount of knowledge, and a set of mechanisms capable of manipulating this knowledge, so as to infer new information. Regarding the knowledge base, Cyc [5], ConceptNet [6], Scone [7], and WordNet [8] are by far the most evolved and successful approaches found in the literature.

To the date, Cyc has got to formalize the largest body of fundamental human knowledge. Nowadays, Cyc Corp is addressing its research efforts to automate the knowledge acquisition, either by interacting with people [9] or by making use of the already asserted knowledge, the natural language understanding, and the knowledge published in the Internet [10]. In contrast to the property system approach followed by Cyc, ConceptNet resorts to the general public for acquiring knowledge. ConceptNet adopts a semantic network structure similar to WordNet. Nevertheless, when compared, ConceptNet claims to hold more informal, defeasible, and practical knowledge. It can also be argued that WordNet should not be listed along with common-sense reasoning systems as Cyc and Scone, for being this just a large database for English lexicon.

Scone is an open-source knowledge based system written in Common Lisp. The main difference with respect other approaches is found in the way how search and inference are implemented. Scone adopts a marker-passing algorithm [11] devised to be run in the NETL machine [12]. Despite the fact that these marker-passing algorithms cannot be compared with general theorem-provers, they are indeed quicker, and most of the search and inference operations involved in common-sense reasoning are supported: inheritance of properties, roles, and relations in a multiple-inheritance type hierarchy; default reasoning with exceptions; detecting type violations; search based on set intersection; and maintaining multiple, overlapping world-views at once in the same knowledge base.

### 2.2. Hardware Architectures for Artificial Intelligence

The NETL machine [12] is the reference hardware architecture for marker-passing operations. This architecture is also the cornerstone for common-sense reasoning, in particular, and graph-based algorithms for Artificial Intelligence in general.

Many early approaches attempted to implement the theoretical artifacts described in the NETL, using technological solutions based on massively parallel and fixed configurations. However, these configurations suffer from scalability issues, mainly due to the fact that every processing element in the data path is associated to a single element from the semantic tree.

One example of a massively parallel architecture is the The Connection Machine (CM). This architecture is composed of thousands of microprocessors, each of which counts on its own memory, behaving itself as a SIMD computer. Microprocessors are arranged, around a network communication infrastructure, in a regular and multidimensional hypercube. In [13], the authors present a software which includes data structures, marker propagation rules and an instruction set for semantic network processing in the CM-2 model. The maximum network size reported in the experimental validation did not exceed the 32K nodes per semantic network, which might be interpreted as a limitation. No further works have been reported in later versions of the CM that would overcome this issue.

The Semantic Network Array Processor [14,15] is another massively parallel platform for high performance and large-scale natural language processing. SNAP employs an extended marker-passing model which makes it also suitable for semantic networks processing. One key characteristic of the SNAP platform is its capability to modify the network model at run time, therefore enabling, also at run time, the dynamic reconfiguration of the communication patterns.

The Parallel Network Wave Machine (PNWM) [16] combines data and control distribution (sending the instructions to be executed to each of the different nodes) to implement a decentralized architecture for high performance semantic network processing. A language called *Wave* has been developed to ease the programming of this data flow computer.

Other architectures put the emphasis on the definition of novel hardware facilities in order to improve the execution time of specific operations in their application domain.

In this regard, the COGnitive ENgine Technologies (COGENT) project presents a processor architecture [17] based on a recirculating parallel computer model with special hardware features to facilitate efficient processing of large graphs. This processor is embedded in a more general framework for cognitive processing that employs intelligent agents. The data (vertices and edge information) is distributed around the local memories of the processing elements or CAGES. This implementation minimizes the cache misses and conflicts in the memory subsystem. The Data Distribution Center is in charge of distributing “*computation*”, firing graph operations on the CAGES. When the processing is done, the Output Filter can be programmed to perform a variety of operations on the produced data (e.g., filtering or coalescing).

A special mention to the IXM2 parallel associative processor [18] is needed. This SIMD architecture is characterized by the use of associative memories for quick access to vertices and fast marker propagation. This strategy is quite similar to the one applied here. Nonetheless, the semantic tree representation and data structures used in our architecture helps to address some of the constrains identified in the IXM2: the use of set hierarchies reduces the opportunities for massive activation and the absence of accumulation and correlation operations on the marker set.

Operational levels of Artificial Intelligence systems benefits from the use of reconfigurable logic. In this sense, the majority of the contributions made, from the reconfigurable logic point of view, are addressed to support common and general mechanisms used in reasoning techniques tasks, such as graph searches. These proposals (or even many others that fall into the category of *reconfigurable computing for parallel computing systems*) could be used just as they are, improving response times of systems. However, it is desirable to access comprehensive solutions for certain application domains based on the use of reconfigurable logic. Comprehensive approaches will enable the same or better (due to customization issues) improvement levels and they will also minimize the integration efforts of the reconfigurable systems.

The work described in this article responds to this philosophy as the one developed by deLomirier *et al.* in GraphStep [19]. In GraphStep specialized processing engines for ConceptNet spreading activation were developed in Xilinx Virtex-2 FPGAs. A Network-on-a-Chip connects all the graph-processing engines together. GraphStep uses regular BRAMs to store the graph data and is able to perform one operation per node and cycle thanks to the pipelining structure of the processing engines.

### 2.3. Scone

The Scone project, led by Scott E. Fahlman at Carnegie Mellon University, represents an open-source knowledge-based approach which, in contrast to approaches such as Cyc [5], WordNet [20], or ConceptNet [21], place the focus not at collecting common-sense knowledge but rather at providing the means for supporting common-sense reasoning mechanisms. The Scone system therefore pays special attention to providing an expressive, easy to use, scalable and efficient approach for accomplishing search and inference operations.

The main difference between this and other approaches lies in the way in which search and inference are implemented. As previously stated, Scone adopts a marker-passing algorithm [11] devised to be run in the NETL machine [12].

One of the main objectives with which Scone was conceived for was to emulate humans' ability to store and retrieve pieces of knowledge, along with matching and adjusting existing knowledge to similar situations. To this end, the multiple-context mechanism implements an effective means to tackle this objective. The multiple-context mechanism also provides an efficient solution by which to tackle a classical problem of Artificial Intelligence, as it is the *frame problem*.

The great potential of the multiple-context mechanism used by Scone can be better stated by using the example described in [11]. Since “Harry Potter World” is quite similar to the real world, a new context, “HPW”, could be created as an instance of the real world (In Scone terminology, “general” is the context node that holds knowledge about the real world, and “HPW” would be an individual node, connected by an is-a link to the “general” node.). Nevertheless, there are differences between these two contexts, such as the fact that in the “HPW” context a broom is a vehicle. This fact can be easily stated in the “HPW” without affecting real world knowledge, in the same way that knowledge of the real world could be canceled so as to not be considered in the “HPW” context. The way in which Scone handles multiple contexts so as to avoid incongruities problems is by activating one context at a time. By doing this, only the knowledge contained in the active context is considered for the reasoning and inference task.

Unless otherwise stated, the knowledge described in a parent context is inherited by the child context. The context itself is also a node and, like the other the nodes, it stores a set of marker-bits. One of these marker-bits is the context-marker. This bit, when enabled, determines the activation of all the nodes and links that are connected to the active context.

Aside from the role that the multiple-context mechanisms play in supporting the possible world theory, the role it plays in describing actions and events is the most important from the Ambient Intelligence point of view. Representing actions and events in Scone simply consists of defining three new contexts, one describing the world before the action or event takes place, another one that represents the state of the world afterward, and finally, one that describes the world properties that hold all along the action performance. In this sense, each of these contexts can be conceived as a possible world, in which the after context world is accessible from the before context goal when the described action takes place. The following example describes a simplified definition of the move event using a syntax similar to that employed by Scone but easier to follow.

NEW–EVENT move : roles  origin is a place  destination is a place  moving–object is a person : throughout  origin differs from destination : before  moving–object is located in origin : after  moving–object is located in destination

In accordance with the aforementioned representation of the move event, the propositional knowledge describing the explicit fact of *Lisa moving*, expressed as Lisa moves, can be also presented as an individual instance of the move event. This individual instance corresponds to the specific occurrence of Lisa moving from the kitchen to the living room.

The declaration of a new instance of the type move event implies that, the new instance named Lisa moves inherits the implicit knowledge of the upper type, the move event. Since the origin and destination of the Lisa moves event have been set respectively to kitchen and *living-room*, the Scone Knowledge-Base can be queried about the location of Lisa at two different time instants or at two different *worlds*, one before the action takes place and another after it takes place. Note how the Knowledge-Base consistency is not affected by that fact that Lisa's location is set to two different places. The use of multiple contexts allows the Knowledge-Base to hold and manage *a priori* inconsistent information in a simple and efficient manner.

**new**–event–indv lisa moves instance –of movethe origin of lisa moves is kitchenthe destination of lisa moves is living–roomthe moving–object of lisa moves is Lisain–context beforestatement–**true**? lisa is in living–room => Noget the location of Lisa => kitchenin–context afterstatement–**true**? Lisa is in living–room => Yes

The answers provided by the Scone system depend on the context that is active at that moment. In this sense, when the active context is set as the before context, the location of Lisa is therefore stated to be the kitchen. Whenever the active context changes to the after context, the location of Lisa is also changed to the living–room.

The most relevant feature of the multiple-context mechanism implemented by Scone is that it supports the construction of a context network along with a context activation scheme. It means that, depending on the desired information, different contexts are activated and deactivated. This feature is particularly important for implementing some reasoning mechanisms for Ambient Intelligence.

## 3. Hardware-Based Reasoning

As it has already been mentioned, the hardware accelerated approach presented here is inspired in the software implementation of the Scone system which, at the same time, implements the marker-passing approach initially devised for the NETL machine. This section starts by providing the reader with a comprehensive overview of the most relevant aspects of the Scone system. Provided the foundations of the NETL and the Scone system, the next subsection is devoted to describing how those have been mapped into hardware implementation decisions.

### 3.1. Scone System Overview

One of the main endeavors of Scone is to optimize the implicit knowledge management, so that even for the worst case scenario the time spent in exploring the facts and properties of the knowledge-base remains constant, regardless of the knowledge-base size. This achievement is basically grounded in the architectural approach adopted by the knowledge-base.

The declarative knowledge kept in the Scone knowledge-base system complies with a semantic network built upon two basic concepts, as they are *nodes* and *links units*. Nodes are the abstractions in charge of representing the conceptual knowledge, whereas links are devoted to representing relational knowledge.

Additionally, both abstractions are implemented by means of structures in which several bits are dedicated to propagate information during the deduction process. This activation mechanism is implemented by means of a bits activation process or, as referred by the Scone literature, by the so-called marker-passing algorithm.

Due to the key role played by the marker-passing algorithm, understanding how such process is implemented in Scone is essential for the hardware optimization pursued by this work. The work in [11] provides a thorough description of how, by means of sequential activations, markers are used to support the inference and search mechanisms that comprise the deductive activity.

Obviously, the fact that the semantic network is implemented by means of a hierarchical structure also plays a relevant role in determining how the deductive search should behave. In this regard, Scone proposes an efficient means of managing duplicated knowledge, as it is the *virtual copy* abstraction. The fact that the different levels of the semantic tree also imply different levels of inheritance can complicate and overload the knowledge-base with information that is already held in it. Scone proposes an efficient solution based on knowledge copies that are virtually present, rather than physically. The most relevant implication of this *virtuality* is the fact that only those nodes that are indeed providing new information about the knowledge already held in the knowledge-base are being physically created. The way in which the remainder properties are inherited is implemented at the level of the marker-passing mechanisms.

### 3.2. A Marker-Parsing Algorithm Overview

The work in [11] describes the insights of the basic maker operations undertaken by Scone. Among all those operations, the upscan one is the *swiss army knife* of the Scone system. Searching, inferencing, and reasoning operations are grounded in the upscan operation. Any query about the relationships, memberships, or properties in which a given node is involved can be stated in terms of an upscanning operation.

This subsection presents the pseudocode for the upscan operation intended to identify the membership relationships in which a given node is involved. In order to do so, as it can be noticed from the pseudocode algorithm, the upscan operation explores all the *is-a* relationships (or membership relations) of a given *node N*, and all the nodes towards which there exists an *is-a* link. The following algorithm is just an extraction of the original one presented in [11]. Please, refer to the original source for further details.



**Algorithm 1** UpscanningIsA(nodeN, markM)
1:markedElements = mark(nodeN, markM)2:**while** isNotAnEmptySet(markedElements) **do**3: isALink = getIsALink(markedElements[i])4: bWire = getBWireOf(isALink)5: **if** isNotMarked(bWire) **then**6:  addToMarkedElements(mark(bWire, markM))7: **end if**8:**end while**


### 3.3. Hardware Implementation Decisions

As mentioned in previous sections, the *marker-passing algorithm* implemented by Scone is one of its main strengths. Briefly, the marker-passing mechanism is mainly devoted to extract the implicit knowledge that it is contained in the *semantic network* by means of propagating pieces of information through the complex web formed by nodes and links. Thus, three main challenges arise when facing the hardware implementation of such a kind of knowledge-base systems: (a) to simplify the representation and storage of the semantic network information; (b) to optimize the memory organization for a fast and efficient implementation of the marker-passing algorithm; and (c) to provide a scalable and distributed architecture that simplifies the task of adding new knowledge while at the same time being capable of performing parallel searches.

**Figure 1.**
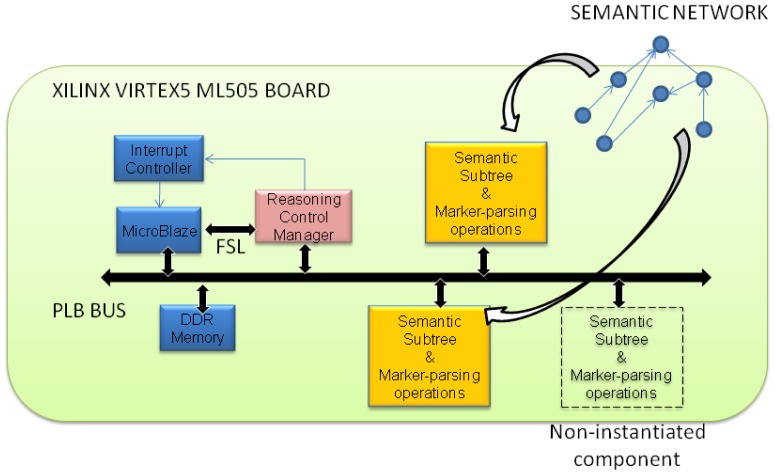
Architecture of the Scone System-on-Chip.

Bearing these goals in mind, a hardware platform, based on the use of FPGAs (*Field Programmable Gate Arrays*) for fast reasoning under the Scone framework has been proposed. In Figure 1, a high level picture of the proposed *reasoning hardware platform* (from now on RHP) is depicted. The system is composed of the following elements:

- A *Microblaze* processor running at a frequency of 125 MHz. There is no other operating system running in the platform but a little software layer (*Xilkernel*) implementing basic services such as task scheduling, thread support and communication with input/output peripherals (*i.e.*, Ethernet interface). The processor runs a small control software routine which is in charge of the synchronization of the operations within the System-on-Chip (SoC) and the communications with a PC. The PC runs a client software that forwards Scone requests to the SoC and also is able to load the semantic tree information in a remote way.
- The *Reasoning Control Manager* (RCM) consists in a coprocessor intended to get the software layer of the system connected with the rest of the RHP. The RCM component is attached to the Microblaze through a *FSL* bus, a point to point serial connector. Applications communicate with the RCM using a FIFO like interface, placing commands in its input FSL channel and retrieving the results from its output FSL channel.
- One or more *Semantic Nodes* (SNs) in which data concerning semantic aspects are mapped into. Each semantic node owns several memories for storing and indexing purposes. They also count on a specialized control logic and data path to implement the supported marker-passing operations. Section 3.6 offers a detailed explanation of the SN internal architecture.
- A DDR2 memory to hold the control program, the kernel software and the configuration bitstream for a *Semantic Node* component template. The bitstream is the information the reconfiguration system of the FPGA needs in order to instantiate new copies of a component.

The device chosen for the implementation of this prototype is a XUPV5 board (http://www.xilinx.com/products/boards-and-kits/XUPV5-LX110T.htm) from Xilinx. It is based on a Virtex5 LX110T chip, equivalent to four million logic gates with run-time partial reconfiguration capability. Our reasoning hardware platform takes advantage of this dynamic feature provided by the FPGA in order to adapt itself to unforeseen scenarios while beings scalable.

For example, the number of SNs instantiated in a given moment depends on the size of the semantic tree capturing the information for the application under execution. From a static point of view, unused SN instances are unnecessarily wasting power consumption. From a dynamic point of view, the proposed platform can freely grow avoiding an oversized initial configuration. If needed, a new SN can be instantiated in a free *partial reconfiguration area* (see white dotted box in Figure 1) in order to expand, for example, the semantic network with a new branch defining a subclass of the *thing* supernode. New entries can be added to the existing SNs through writing operations performed on local memories. The work proposed here resorts to the infrastructure for dynamic reconfiguration management for Xilinx platforms developed by Dondo *et al.* [22].

Another scenario that is worth being mentioned refers to the fact that it is possible to face a run-time rearrangement of the semantic tree data among the SNs. For optimization purposes (see Section 3.5) it would be necessary to split one branch of the semantic tree (actually stored in one SN) and consequently more SNs will be instantiated to hold the resultant subtrees.

### 3.4. Overall System Operation

This section is aimed at presenting an overview of how the RHP works at system level, before getting into the implementation details of the individual Semantic Nodes. From the user's perspective, the presence of the RHP is totally transparent since it is remotely used through the standard Scone interface.

The board with the RHP is connected to a PC using the Ethernet interface. The PC runs a modified version of the Scone system that is able to communicate with the control software running in the RHP. Every time a reasoning operation is requested, it is forwarded to the board, encapsulated in a UDP packet. The control software receives the message and translates it into commands that are understood by the RCM and the SNs in the platform. Only the Scone's java libraries which implements the search and inference functionality have been extended to support this feature. Therefore, the way the user interacts with Scone remains unchanged.

The other extension to the Scone platform is the addition of a new set of commands: (a) to control the creation and disposal of new SNs in the RHP and; (b) to deploy the semantic tree data. Table 1 provides a summary of the new functionality in Scone.

**Figure 2.**
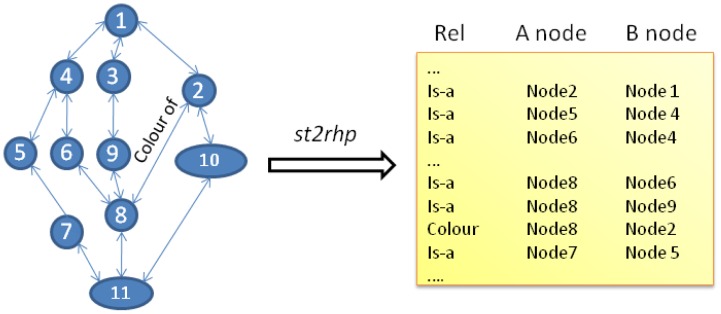
The *st2rhp* command translates the semantic tree data from the computer format (**left**) to the tabular structure used by the RHP (**right**).

Since the way in which the semantic tree data and structure is codified in the RHP differs significantly from the way how same information is stored in the computer, a pre-conversion stage is required. The *st2rhp* command listed in Table 1 is devoted to perform that duty. In the RHP, the network of nodes and pointers that interconnect them are translated into a tabular format. Adopting a tabular form guarantees the optimum management and operation simplification of the data structure handled by the hardware platform. Internally, each node entity of the semantic tree is translated into a unique integer value which clearly identifies it in the system. Relationships among nodes are translated into table entries qualified by the type of relation (*i.e.*, *is-a*) and the direction of such relation (*i.e.*, *A-node* or *B-node* using Scone terminology). Figure 2 graphically represents the process. The tabular representation used in our approach is quite common when facing the problem of implementing pointer-based data structures from software to hardware-friendly and equivalent constructions that have to be operated by logic [23,24].

After the UDP packets arrive at the RHP, the Scone operations are codified as 32 bit commands to be processed by the RCM one at a time. The RCM issues the corresponding bus transactions and waits for its completion before signaling the software such condition by means of an interruption. Table 2 summarizes the supported commands and the *instruction format* for each kind of operation.

The RCM propagates a command to all Semantic Nodes that are active and connected to the system bus at that moment. The RCM counts on a memory (the SN directory) in which the information required to address SNs in the system is stored. At the lowest level, whenever the RCM receives a 32 bit word command it triggers a bus transaction, per SN directory entry, with the command value and the address retrieved from the SN directory. For *upscan* and *downscan* operations, the command received by one SN can be, in practice, discarded if the identifier of the *Starting Node* is not present in its *Semantic Tree CAM* (Section 3.6).

In order to control the distributed execution of an operation, each SN core must notify back to the RCM the *initiation* and *finalization* of the local activity. This is done by means of special bus write transactions signaling such execution conditions to the RCM. To this end, the RCM holds another RAM in which the RCM scoreboards the start/end notifications. When all the pending works have come to an end, the RCM is ready to pop the next command out of the input FIFO.

In the case of a *get_nodes* command, every SN sends to the RCM the list of node identifiers marked together with the *finalization* condition. The complete list of nodes is temporarily stored in the output channel FIFO. The software is responsible for reading those values.

The HW/SW and HW/HW communication relies upon the OOCE [25] interfacing infrastructure, mechanisms and protocols. The Object-Oriented Communication Engine (OOCE) is a system-level middleware particularly designed for SoCs which provides a high-level and homogeneous view of the system components based on the Distributed Object paradigm. Communication between components is abstracted by means of a HW implementation of the Remote Method Invocation semantics and all the SW and HW adapters are automatically generated from functional descriptions of the components interface. The resulting communication infrastructure simplifies the integration effort required and makes the embedded software more resilient to changes in the HW platform.

### 3.5. Optimized Marker Propagation

*Upscan* and *downscan* operations are the most costly ones in terms of required time to be completed. Unlike the *get_nodes* and *clear_markers* commands, which can be triggered in all SNs concurrently, these tree searches cannot be parallelize because of the preceding dependencies inherent to the tree topology. However, it would be desirable to figure out a method to exploit concurrency in the RHP. We propose the use of two simple techniques in order to reduce the overall latency of *upscan/downscan* executions in our platform: (a) horizontal or *branch partitioning*; and (b) vertical or *depth partitioning* with the help of *forward links*. These methods are based on a smart spatial distribution of the semantic tree data among several SNs. Let us illustrate how the marker propagation process is improved through a series of simple examples.

**Figure 3.**
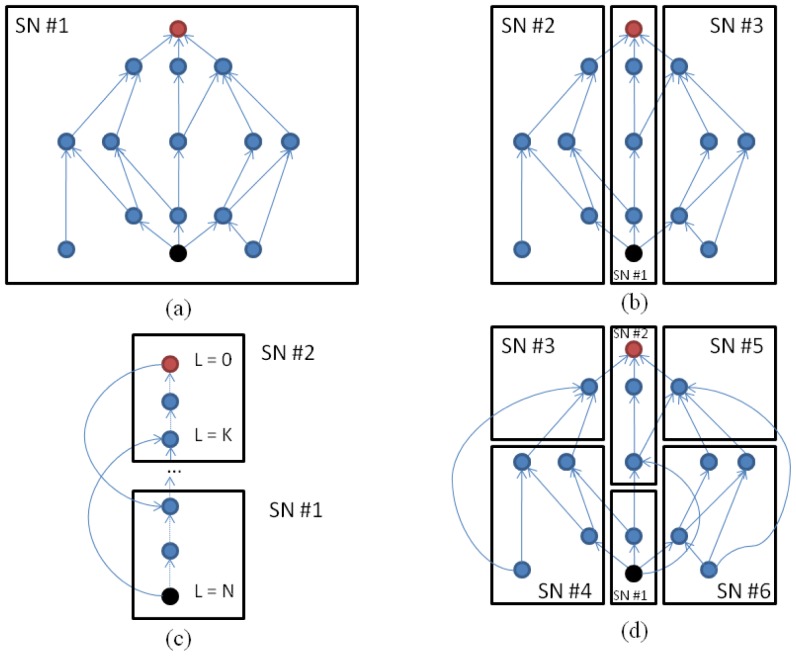
Four possible distributions for a semantic tree: (**a**) single SN; (**b**) branch partitioning; (**c**) vertical partitioning with forwards links; and (**d**) arbitrary combination of both.

Figure 3 represents a schematic picture of a simple semantic tree with four possible distribution configurations among one or more SNs. The first case (Figure 3(a)) is the non-optimized version in which the whole data tree is stored in a single SN. Although individual SNs perform marker operations quite efficiently, due to the specialized hardware architecture, this configuration prevents the concurrent propagation of markers and searches. This issue is analyzed with further detail in the next subsection. Within a SN, tree traveling through the links and nodes is performed sequentially (see Section 3.7), in the order specified by a breadth-first algorithm. Thus, the necessary time to complete one of these operations mainly depends on the total number of links that must be visited. This number is related to the depth and width of the tree.

Now, let us imagine that we start an *upscan* or *downscan* operation from the black and red nodes respectively on the same tree. If the tree is broken down into three parts (the criteria followed here consists in detaching the main branches of the tree) and each part is sent to different SNs, the propagation can be done in parallel. Figure 3(b) depicts this scenario. Although the maximum depth of the individual subtrees is equal to the former tree, the total number of links to be traversed for each one is reduced, and therefore the total time to complete the propagation is reduced. In this example, the activation of the operations in all the SNs involved in a search task takes place this way:

- First, the RCM requests the initiation of the upscan or downscan commands to all the active SNs.
- Each SN looks into its own tree data and determines whether it is necessary to start a marker propagation activity or not. Since one node is owned exclusively by one SN, only one SN is activated (in our example, *SN #1* for both cases).
- Once the propagation process reaches an *edge link*, the SN requests the *target SN* to initiate the same command. An edge link represents a relation between two nodes in the semantic tree where one of them does not resides in the same SN. Therefore, in order to be able to continue traveling the semantic tree it is obliged to store the location of such node (which in practice means to register the base address of the SN core that owns the node at the end of the link). When the SN control logic finds an *edge link*, the same operation is triggered either in the *A-node* or the *B-node* depending on the specific operation under execution. Back in our example of Figure 3(b) after marking the red or black node, two upscan or downscan operations will be passed on to *SN #2* and *SEN #3*. From this point marker propagation runs in parallel in SN #1,#2 and #3.

Another possible optimization to accelerate marker-passing algorithms in RHP is the one represented in Figure 3(c). In this case, the branch is split *vertically*. This technique reduces the total depth of the just partitioned branch. The depth reduction also supposes a reduction of the time involved in visiting all the nodes. However, the sole division of the data structure does not have the desired effect. In order to early activate a parallel search or an upwards or downwards propagation, it is necessary to add extra knowledge to the semantic tree in the form of *forward links*.

A forward link is an edge link that captures the transitive property of a given relation. Contrary to edge links, forwards links are not present in the former semantic tree but are added after the vertical partitioning is done. In the case of Figure 3(c), two new forward links were added to the original semantic tree. The first one (from the red node in level 0 to the blue node in SN#1) enables a soon start of marker propagation in SN#1 while it takes place in SN#2 at the same time. The second forward link (from the black node in level *N* to the blue node in level *K*) plays the same role but for upscan operations.

In both cases (vertical and horizontal partitioning), inter-SNs propagations can reach a node that has been previously visited. In this case, to reduce the number of internal operations on the local memories, the SN tracks those nodes (setting the *visited* flag for such entry) for an operation. The SN control logic discards such operations since it is able to detect overlapping, cutting the propagation of any marker that has been set/clear before.

Finally, arbitrary combinations of these techniques can be done (see Figure 3(d)). It is out of the scope of this work to assess the algorithms or approaches that would result in the best partitioning solution for a given semantic tree.

### 3.6. Architecture of the Semantic Nodes

**Figure 4.**
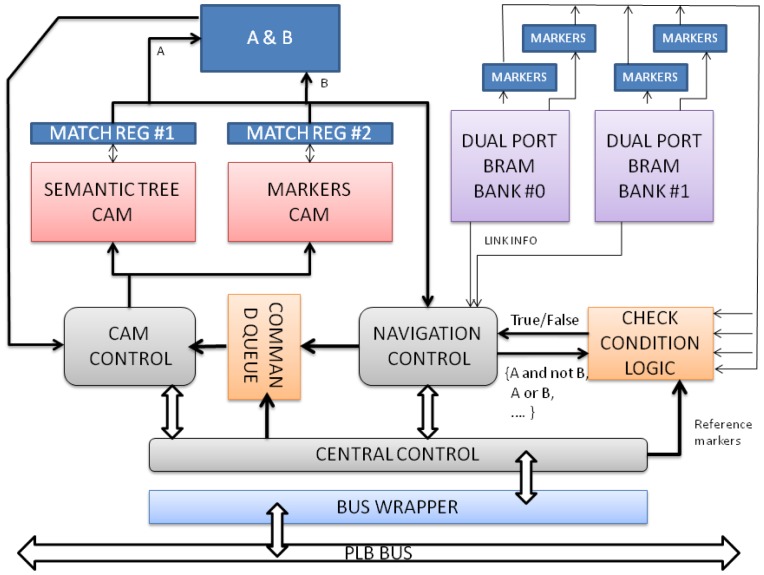
SN internal block diagram.

The section describes the internal architecture of the individual Semantic Nodes, as hardware cores attached to the system bus. SNs are in charge of supporting the execution of the RHP commands (Table 2) undertaken upon the fragment of the semantic tree assigned to each of them. RHP commands are translated into bus write transactions by the RCM, where the data bits encapsulate the 32 bit representation of the operation. Figure 4 shows a high level diagram block of the main components that conform to the SN architecture. A brief explanation of each of these components can be found underneath:

- **Bus wrapper**. It is the OOCE adapter to the bus that is in charge of isolating the core logic of the SN from the implementation details of physical protocol bus. This makes it easy to port the component to other bus infrastructures and interface to the software infrastructure.
- **Central control**. This component is in charge of managing the operation execution and the synchronization with other control components.
- **CAM control**. It is responsible for interpreting the actions to be performed on the CAM (*Content Access Memories*) memories: *match* and *write*.
- **Command queue**. This component is implemented as a FIFO in which CAM control commands are stored. The *match* command checks whether an input bit pattern matches any content in the CAM. The *write* command modifies the content of the CAM.
- **Content Access Memories**. They are used to index the Block RAMs (BRAMs). Due to technological restrictions, the maximum addressable content in these memories is 4096 entries each. Approximately 20 SNs are needed to store a semantic tree of 10^6^ elements (including nodes and relations). There are two types of CAMs in the design:
  – The *Semantic Tree CAM* (STC) is used for navigation purposes. Each entry in this CAM represents a relation between two entities in the semantic tree as it is explained below.
  – The *Markers CAM* (MC) maintains a copy of the marker bits for each relation which applies to both nodes at the ends. This CAM is necessary to implement conditional selections (*i.e.*, all the predecessors of a node that has markers M1 and M2 activated): one pattern is given to the Semantic Tree CAM and the other to the Markers CAM.
- **Match registers**. Temporal registers where the hits found in the CAM are stored. The output of the CAMs is configured not to be coded. Therefore, *one hot coding* is used instead, which simplifies the control process of the operation.
- **Navigation control**. Given a mask of hits, this component schedules the read/write operations on the Block RAMs. Also, it feeds the command queue with new operations. For example, in a downscan operation, the CAMs are used to point out where the successors of a node are in the BRAMs. The node numbers are read from the BRAMs and the navigation control asserts new match command to the queue, this time to find the successors of the child nodes and so on.
- **Dual port Block RAMs**. A replica of the Markers and Semantic Tree CAMs is stored in these memories together with status and property bits to control the navigation/propagation process (*i.e.*, the external link flag). The data is interleaved among the two memories banks in order to perform up to four memory operations simultaneously.
- **Check condition logic**. It is a combinational configurable (through a select operation port) block that implements the logic comparison between the marker bits in memory and the marker masks in a *get_nodes* command.

### 3.7. Basic Reasoning Operations

*Upscan* and *downscan* operations are basically conceived as a way to traverse the tree structure in order to activate a set of marker bits. For that reason, the way how semantic tree relations are represented in the different SN memories has an important impact on performance. Each word in the STC maintains the following information for a semantic tree link: identification of the type of relation (*i.e.*, is-a, equals,…), the *A node* identifier and *B node* identifier. In Scone, the A node plays the role of *son* in a relationship whereas the B node is the *father*. With this simple configuration fast retrieval operations can be performed, for example, “*find all the predecessors of this node for a relation*” or its complementary one with the successors. In only one cycle, given the appropriate search mask at the input, the CAM output register provides the memory addresses that satisfy that condition.

**Figure 5.**
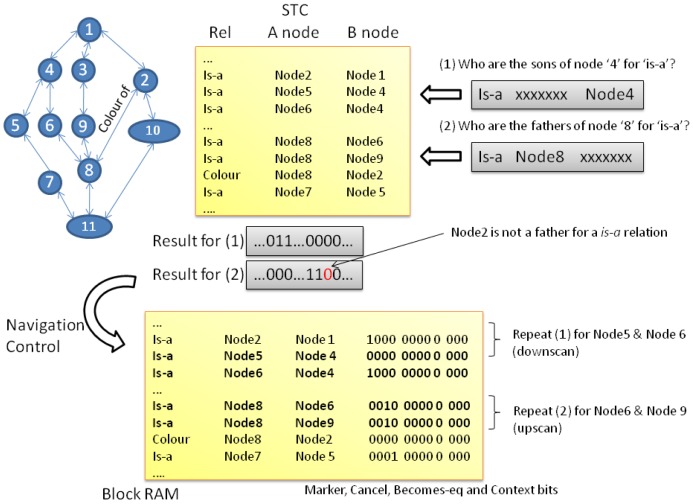
Memory content and basic tree search functionality.

In Figure 5 the reader can find two simple examples that help to understand the whole process. First, the semantic tree info was converted (*st2rhp* command, Table 1) into the RHP format that can be seen in the upper part of the figure. The BRAM content which is presented in the lower part of Figure 5 is equal to the ST CAM content plus the marker bits. Other control bits such as the *cancellation, becomes-equal, visited and context* bits are also present in the BRAM. These bits are interpreted by the navigation control module to determine the next set of nodes to visit in the next iteration. For basic reasoning with no exceptions, only the marker bits are significant.

In *upscan* and *downscan* operations, a marker is propagated upwards or downwards iteratively through the tree hierarchy following a specific relation type. Marker propagation actually means to set to one the corresponding marker bit both in the BRAM and the MC. Recall that a copy of the marker bits is maintained in the MC so it is possible to select only those nodes that have the proper marker bits activated (*logic* and between the STC output register and the MC).

Let us focus in case (1) within Figure 5. In this case we want to get the nodes that are of the same class of *Node 4*, which is equivalent to asking about the sons of *Node 4*. At the lowest level that means search for a bit pattern (grey box) in the CAM that has defined the type of relation (in this case *is-a*) and the parent node. Every entry that matches such pattern codifies a relation in which *Node 4* is the father (B-node field). The B-node field, thus, will have the identifiers for the son nodes. The STC output is a bitmask in which one bit represents a memory position in the CAM and the BRAM. If the bit *N* is set to 1, it means that entry *N* satisfies the primary condition (to be son of). Then, the navigation control for every active bit performs two memory operations on the selected address: (a) it reads the memory to get the data (CAM memory content cannot be read, so it is not possible for the control to determine the node identifiers that are *sons of*) and push a new command in the *command queue*. In our example, it will generate two new commands: (a) it asks who are the sons of nodes 5 and 6, (b) it writes the new value for the marker bit that is being propagated.

### 3.8. Advanced Features: Cancelation, Virtual Copies and Contexts

Advanced Scone features have also been considered in this work. This exposition starts by describing the *cancellation mechanism*. This mechanism is intended to model exceptions of the form “*all birds can fly but penguins can not fly although they are birds*” (This is the example provided by S.E. Falhman in [11]). To model this situations, Scone uses special markers (cancellation bits) that block the propagation of another marker. In the hardware implementation, first *cancellation links* are explored before initiating the propagation to the next level. A cancellation link is a regular link that holds complementary meaning (for example, a *is-not-a* represents the cancellation link for a *is-a* relation). Second, a propagation of the cancel bit starts by setting to one the cancel bit in the STC and BRAMs memories (this propagation is done by using the regular propagation procedure). Third, the former *upscan*/*downscan* operation can proceed but this time, if the corresponding cancel bit is activated for a link, the navigation control will stop the propagation and will not include that node in the *nodes to be processed* list.

*Virtual copies semantics* in Scone avoids the unbounded growth of the semantic data when trying to model complex relations. Typically, those relations involved in ternary relations to model different roles a node can play in different relations. For instance, one person can play the role “daughter” and “mother” in two distinct families. Without the existence of Scone's virtual copies semantics, the information to model such scenarios should be replicated. Generally speaking, the goal of those ternary representations is to enable reasoning paths that were originally unavailable, because a marker cannot cross different relation domains (e.g., if Mary *is-a* Mother in Smith Family and Ashley *is-a* Child in Smith Family and Mothers *love* Children, then Mary *loves* Ashely). The implementation of virtual copies is realized by means of *qualified* links that represent such ternary relations. In hardware, those relations are taken apart in a separate CAM, the *Virtual Copies CAM* (VCC), which was not depicted in Figure 4 for the sake of simplicity. The standard propagation mechanism is altered this way:

- If the node participates in any ternary relation, then the *becomes-equal bit* is set to one in every node involved in a ternary relation with the same owner.
- Proceed with the standard propagation procedure.
- Continues propagation for every node with the operation marker bit and the *becomes-equal bit* activated.

Finally, context modeling in the RHP is sketched out. As stated in Section 2.3, the potential of Scone's multiple context modeling resides in the fact that semantic information can be reused from one context to another just by adding the context specific information or canceling facts. Following the HPW example, it is necessary to state that a broom *is-a* vehicle and also *is-a* flying thing. That could be done only by adding the necessary links to the real world context. However, such links are only meaningful in the case the HPW is activated. Context activation is handled in the same way Scone proposes. We store a *context* bit mask in the memories, if the right bit is set to one then the link (STC entry) is considered in the reasoning operations. Context activation must be performed beforehand, for such purposes the context-specific semantic data is loaded in the SNs, each entry with an associated context bit already activated. To simplify the reasoning procedures, the entries with all their context bits set to zero are said to belong the “general context” and participates in all the operations.

## 4. Experimental Results

The evaluation of the RHP presented here needs to be performed in comparison with the “only software” implementation of the Scone system. The set of selected tests reproduces the benchmark designed by Scott E. Falhman in [11] to demonstrate how fast is Scone for the main inference operations.

As in [11] we have used a synthetic knowledge base of half million elements (counting nodes and links). Since the total size of the knowledge base exceeds the maximum data a single semantic node can maintain, it has been spread among a total number of 40 cores, which represents a fairly large and complex system. The partitioning strategy employed combined both a vertical and a horizontal approach with the only goal to evenly distribute the number of nodes among the total SN cores available.

The computer used to obtain the reference execution times was a Dell XPS 8300 workstation with 8 GB of DDR3 memory, an Intel i7-2600 quad-core processor running at 3.4 GHz. The operating system installed is a 64-bit GNU/Linux (kernel version 2.6.16). Due to space constrains, Table 3 shows the results for the test bed executed on both the workstation and the FPGA based prototype of the RHP.

The measured time for the first test that loads the semantic tree info is nearly twice in hardware than in software. Notice that the hardware time for this test includes the time invested in loading the semantic tree information into the PC memory, the conversion time of the data to the RHP format, the transmission to the board through the 100 Mb Ethernet interface and the time to write the values in the FPGA local memories of the SN cores. This is the reason for such difference in the preparation times.

The rest of tests do not include the configuration times. The main conclusion studying these results is that with the support of our RHP it is possible to improve the overall performance of the system in factor that ranges between 5 and 10 times faster.

Concerning the hardware resources consumed by one instance of a Semantic Node, the synthesis of one element needs of 1196 FPGA slices (7% of the total) and 4 Block RAMs (36 Kbit size each). The core runs at the maximum frequency for the system of 125 MHz.

The hardware accelerated implementation presented here has paid special attention to improving the ternary relationships that emulate the Scone *virtual copies* abstraction. Further tests need to be carried out in both implementations in which virtual copies plays a key role, so that a better comparison of both implementations can be provided.

## 5. Conclusions

Ambient Intelligence systems are expected to wisely supervise the activities that are taking place in the context, independently of application domain (home, entertainment, assisted living, *etc.*). In order to do so, these systems have to understand the ongoing situations by interpreting the events captured from the context. However, it is a fact that the majority of the systems proposed to date fail in achieving flexible solutions capable of reacting to unforeseen situations, for example. The fact that those systems have been designed in a condition-reaction fashion made them unsuitable to meet the requirements of what an Ambient Intelligent system should be.

This work identifies the lack of common-sense knowledge as the main reason why Ambient Intelligence systems have failed to succeed. For that reason, this work adopts the axiomatic fact that Ambient Intelligence should follow the approach of artificial intelligence researchers addressing the problem of achieving intelligent systems. In this sense, it is necessary to provide an architectural solution capable of dealing with the vast amount of knowledge that comprises the common-sense knowledge. Also, efficient mechanisms to handle such information have to be provided in order to support the extract new information out of the information already asserted to the knowledge base, based on search and inference strategies.

This work has been mainly devoted to propose a hardware accelerated implementation of the Scone common-sense knowledge-based system. The ultimate goal of this implementation is to provide a feasible means upon which context modeling and reasoning tasks can be undertaken at run time.

The main contributions of this work can therefore be summarizes as follows:

- It points out that the lack of common sense is the main reason why Ambient Intelligence systems have not been a reality as it was foreseen more than a decade ago.
- It states the need for a system capable of handling common-sense knowledge.
- It proposes a hardware-based system for commons-sense system, in which the Scone system's most powerful mechanism has been translated into hardware implementations.
- It validates the hardware-based solution with a set of experimental tests that evidence the promising opportunities that this approach might bring to the field of Ambient Intelligence systems.

## Figures and Tables

**Table 1.** New commands added to Scone to integrate with the proposed Reasoning Hardware Platform.

**Command**	**Function**
*newsn*	Creates a new instance of a SN core. The control software in the board loads the bitstream from memory and configures a free reconfigurable area.
*removesn(idsn)*	Disposes a SN instance. The data in the SN is lost.
*select*	Selects a portion of the semantic tree that will be used further by other commands. The specification of the parts to extract is made in term of maximum number of branches to include, maximum depth of the resulting subtree, *etc.*
*st2rhp*	Takes a selection from the *select* command and converts it to the internal format understood by the RHP. The result is sent to the RHP using the *send* command.
*send(data,idsn)*	Transmits data in semantic tree RHP format to the selected SN.
*clear(idsn)*	Deletes the memory content of the selected SN.

**Table 2.** Supported commands by the Reasoning Hardware Platform.

**Name**	**upscan/downscan**	
Description	Mark the *StartingNode* with *MarkerField* and propagates it through *RelationType* in the specified direction	
Field Name	Description	Bits
OPCode	Operation code (00/01)	2
StartingNode	Entity ID the upscan process starts off	20
MarkerField	Boolean mask indicating the indexes to activate	8
RelationType	Type of link	2
Description	Retrieve all nodes that matches the condition specified	
Field Name	Description	Bits
OPCode	Operation code (10)	2
Condition1	*is-set* (1),*is-clear* (0)	1
MarkerField1	Boolean mask indicating the indexes that must satisfy *condition1*	8
**Name**	**get_nodes**	
BooleanOp	Operation to relate both conditions. 00 *nothing*, 01 *and*, 10 *or*, 11 *and-not*	2
Condition2	*is-set* (1),*is-clear* (0)	1
MarkerField2	Boolean mask indicating the indexes that must satisfy *condition1*	8
**Name**	**clear markers**	
Description	Set to zero all marker bits	
Field Name	Description	Bits
OPCode	Operation code (11)	2

**Table 3.** Mean execution time of relevant Scone reasoning operations in the HRP and Dell workstation. 500 executions of each test were carried out.

**Test**	**SW Time**	**HW Time**
Load the semantic tree (500K elements, no checking)	109 s	210 s
Downscan the semantic network tree (500K elements)	2.7 s	0.41 s
Check the type of a given individual	0.23 ms	0.04 ms
Mark & intersect 2 sets with 10K members, one winner	20.71 ms	2.88 ms
Mark & intersect 3 sets with 10K members, one winner	36.9 ms	5.59 ms
